# Palm Kernel Cake in Diets for Lactating Goats: Qualitative Aspects of Milk and Cheese

**DOI:** 10.3390/ani11123501

**Published:** 2021-12-08

**Authors:** Fernanda G. Ferreira, Laudí C. Leite, Henry D. R. Alba, Bruna M. A. de C. Mesquita, Stefanie A. Santos, Manuela S. L. Tosto, Marion P. da Costa, Douglas dos S. Pina, Layse A. Gordiano, Arielly O. Garcia, Pedro H. S. Mazza, Gleidson G. P. de Carvalho

**Affiliations:** 1 Department of Animal Science, Federal University of Bahia, Av. Adhemar de Barros, 500, Ondina, Salvador 40170110, Brazil; fernandagazarufrb@gmail.com (F.G.F.); henry.ruiz@ufba.br (H.D.R.A.); stefanie.alvarenga@ufba.br (S.A.S.); mtosto@ufba.br (M.S.L.T.); marioncosta@ufba.br (M.P.d.C.); douglas.pina@ufba.br (D.d.S.P.); lay_gordiano@hotmail.com (L.A.G.); pmazza@ufba.br (P.H.S.M.); 2 Department of Animal Science, Universidade Federal do Recôncavo da Bahia, Cruz das Almas 44380000, Brazil; laudi@ufrb.edu.br; 3 Institute of Agricultural Sciences, Universidade Federal de Minas Gerais, Montes Claros 39404547, Brazil; brunacarvalho@ufmg.br; 4 Department of Animal Science, Universidade Federal de Viçosa, Viçosa 36570900, Brazil; arielly.garcia@ufv.br

**Keywords:** byproduct, dairy goat, fatty acids, ruminant nutrition, sensorial analysis, small ruminant

## Abstract

**Simple Summary:**

Feedlotting lactating goats is a strategy to improve their productivity and the quality of their milk and dairy products. However, feedlotting is associated with increases in production costs, due mainly to the concentrate component of the diet. The use of agro-industrial byproducts allows the reduction of feed costs by replacing costly ingredients, as the former are more easily accessible. We tested the dietary inclusion of palm kernel cake (PKC), a byproduct of the biofuel industry, at the levels of 0, 80, 160 and 240 g kg^−1^, to evaluate its impact on the qualitative aspects of milk and cheese. The inclusion of up to 80 g kg^−1^ PKC is recommended for the diet of goats whose milk will be used in the production of Minas Frescal cheese.

**Abstract:**

We investigated the effect of including palm kernel cake (PKC) at the levels of 0, 80, 160 and 240 g kg^−1^ in the diet of lactating goats on the quality and sensory parameters of Minas Frescal cheese. Twelve goats were used in a triple 4 × 4 Latin square design. The dietary addition of PKC was associated with a reduction in moisture (*p* = 0.004), which compromised the cheese yield (*p* = 0.030). The ether extract content showed a quadratic response. There was a decrease in caproic (*p* = 0.014), caprylic (*p* = 0.011), capric (*p* = 0.003) and palmitic (*p* = 0.049) acids and an increase in lauric (*p* = 0.012) and myristic (*p* = 0.02) acids. Monounsaturated fatty acids increased (*p* = 0.008), whereas the ratio of polyunsaturated to saturated fatty acids (*p* = 0.022) and thrombogenicity index (*p* = 0.023) decreased. Principal component analysis indicated that control treatment and the treatment with 80 g kg^−1^ PKC were the most acceptable to the cheese tasters. The use of palm kernel cake is a good alternative for lactating goats when added to the diet at levels up to 80 g kg^−1^.

## 1. Introduction

Goat milk and cheese have nutritional qualities relevant to human health due to the similarity between this and human milk, as well as smaller fat globules and a lower proportion of allergenic proteins when compared with cow milk [1]. However, goat farming is mainly practiced in rural areas, in extensive production systems [2]. In these systems, these animals are fed diets based on highly fibrous pastures of low nutritional quality, which limits their production performance [3].

Feedlotting is an important strategy to provide the nutrients necessary for the maintenance and production of animals. This practice allows increases in yield [4] and prioritizes the use of concentrate diets. Nonetheless, feeding is the costliest factor in animal production, accounting for up to 70% of total costs [5]. As a strategy to reduce production costs, unconventional ingredients are used to replace ingredients of high commercial value, such as soybean meal and ground maize.

Among unconventional feed ingredients, byproducts have successfully been used to reduce the disposal of these materials into the environment. In addition, the efficient use of by-products in ruminant diets can result in the production of foods of high biological value for humans (e.g., milk and meat) [6]. The oil palm (*Elaeis guineensis*) is a plant of South African origin that stands out for its high bioenergetic potential and ease of cultivation, as it adapts well to different soil–climatic conditions [7]. Palm kernel cake (PKC) is obtained after the oil is extracted through pressing. The chemical composition of the cake includes 96.6–105.6 g kg^−^^1^ ether extract (EE), 143.4–169 g kg^−^^1^ crude protein (CP), and 599–656.3 g kg^−^^1^ neutral detergent fiber (NDF) [8,9,10]. These nutritional characteristics are promising for the formulation of animal diets. In a study including the sensory evaluation of meat from goats fed diets containing PKC, greater sensory acceptance was achieved with the highest inclusion levels (240 and 360 g kg^−^^1^ dry matter (DM)) [11].

Thus, the use of PKC in the feeding of lactating goats is not expected to adversely affect the sensory aspects of milk and cheese, but rather to improve the quality of these products.

In this scenario, this study was developed to examine the effect of including PKC in the diet of dairy goats, at the levels of 0, 80, 160 and 240 g kg^−1^ DM, on the dry matter intake, fatty acid profile, milk yield and composition, and the sensory quality of their milk and Minas Frescal cheese, made from this milk.

## 2. Materials and Methods

### 2.1. Ethics Committee and Experiment Location

The experiment followed animal welfare rules; hence, the project was approved (approval no. 73/2018) by the Ethics Committee on the Use of Animals (CEUA) at the Federal University of Bahia (UFBA). The experiment was conducted in the goat-farming section of UFBA, located in the municipality of Entre Rios—BA, Brazil (11°56′31″ S, 38°05′04″ W, 162 m above sea level).

### 2.2. Animals, Experimental Design and Management

Twelve multiparous lactating goats were used in a triple Latin square experimental design, consisting of two squares with four Saanen goats and one square with four Anglo-Nubian goats (multiparous, average weight of 46.9 ± 9.4, average of 100 days in milk and average production of 0.7 kg day^−^^1^).

The experiment lasted 71 days, which included 15 days for the animals to acclimate themselves to the facilities, milking management and diets. The remaining 56 days were divided into four experimental periods of 14 days each, of which ten days were used for the animals to adapt to the treatments and four for data collection.

The diet was formulated according to the NRC [12], to meet the requirements for maintenance and milk production. The experimental treatments (Table 1) consisted of the inclusion of PKC at the levels of 0, 80, 160 and 240 g kg^−^^1^. The diets were supplied as a total mixture, twice daily (8 a.m. and 3 p.m.). A forage:concentrate ratio of 40:60 was adopted, with maize silage used as forage.

The goats were housed in individual pens with an area of 1.5 m^2^, which were equipped with a drinker and a feeding trough. Water was provided in adequate quantity and quality, and feed was supplied with daily adjustments to allow around 10% orts.

Milking was performed at 7 a.m., after pre-dipping the teats with a 0.5% glycerin iodine solution. After milking, post-dipping was performed by immersing the teats in a 0.5% glycerin iodine solution. Hygiene measures for milkers were followed and the place and utensils used for milking were cleaned.

### 2.3. Intake

Intake was calculated as the difference between the amount of the component present in the feed supplied and in the orts.

### 2.4. Chemical Analysis

During the experimental period, samples of ingredients and orts were collected and dried in a forced-air oven at 55 °C for 72 h. Once dried, the samples were divided into two portions that were either ground in a Wiley knife mill into 1 mm particles for chemical composition analysis, or into 2 mm particles to determine the neutral detergent fiber (NDF) content. These samples were then used to measure the DM (934.01), ash (930.05), CP (981.10) and EE (920.39) contents, following the methodology proposed by the Association of Official Agricultural Chemists (AOAC) [13].

Neutral detergent fiber and acid detergent fiber (ADF) were determined as proposed by Van Soest et al. [14], with the adaptations described by Mertens [15]. Neutral detergent fiber corrections for ash and protein (NDFap) were performed according to Sniffen et al. and Licitra et al. [16,17], respectively. Lignin was determined, according to the AOAC method 973.18 [18], by immersing the ADF residue in a 72% sulfuric acid solution.

Indigestible neutral detergent fiber (iNDF) was determined by the in situ incubation of samples inside non-woven fabric (“TNT”) bags weighing 100 g m^2^, following the methodology described by Valente et al. [19]. Potentially digestible neutral detergent fiber (pdNDF) was determined as the difference between neutral detergent fiber corrected for ash and protein (NDFap) and iNDF.

### 2.5. Milk Composition

Milk production was determined per animal and per day during the last four days of each experimental period. After collection, milk was measured using a graduated measuring cylinder of one liter in volume.

Milk samples were collected and a portion of each was placed in a plastic bottle containing the preservative 2-bromo-2-nitropropane-1,3-diol (bronopol) for the analysis of protein, fat, lactose, urea nitrogen and total solids, using the Bentley-2000 infrared analyzer, as well as somatic cell count, using the Somacount-500 instrument. These analyses were performed at the laboratory of the Clínica do Leite at ESALQ/USP, in Piracicaba-SP, Brazil.

To determine the values of the milk components in g day^−1^, the percentage of each component (fat, protein, lactose and total solids) was multiplied by the volume of milk produced (g day^−1^).

### 2.6. Milk Fatty Acid Profile and Fat Quality Analysis

Milk samples were stored in airtight containers and kept frozen at −20 °C until the moment of fatty acid profile analysis. The milk was slowly thawed in a refrigerator and homogenized, and an aliquot (10 mL) was collected and centrifuged. After centrifugation, the supernatant was subjected to a fat extraction procedure with the organic solvent hexane. For the methylation of fatty acids, a basic catalyst (sodium methoxide) and an acid catalyst (acetyl chloride) were used in a two-step methylation process [20].

Fatty acid methyl esters were quantified by gas chromatography (Focus GC-Thermo Scientific, Thermofisher, São Paulo, Brazil) with a flame ionization detector (CG-DIG) and an SP-2560 capillary column (Supelco, 100 m × 0.25 mm × 0.2 µm). Hydrogen was used as carrier gas at the rate of 1.5 mL min^−1^. Detector and injector temperatures were fixed at 250 °C. The initial temperature of the column was set at 70 °C, held for 4 min, raised to 175 °C at a rate of 13 °C per minute, held for 27 min, and finally raised again up to 215 °C at a rate of 4 °C per minute and held for 31 min [20]. The fatty acid methyl esters were identified based on the retention times of the FA 275 standard (GLC-674, Nu-Chek Prep Inc., Elysian, USA).

The obtained results were used to calculate the total saturated (SFA), monounsaturated (MUFA) and polyunsaturated (PUFA) fatty acids and the ratio of omega-6 (n-6) to omega-3 (n-3) fatty acids.

The indices that indicate the quality of milk fat were calculated from the equations proposed by Ulbrich and Southage [21].

### 2.7. Cheese-Making Process

Milk was collected in the last four days of each experimental period for the production of Minas Frescal cheese. The milk was weighed, sieved, and stored individually in airtight containers at −20 °C.

Later on, the milk was thawed in a refrigerator and sieved again. For the manufacture of Minas Frescal cheese, the sanitary norms set forth by Ordinance no. 326 of the Brazilian Ministry of Health were followed to ensure its safety and quality for human consumption [22].

The milk was pasteurized at a temperature of 60 °C for 30 min and then cooled in an ice bath until it reached 38 °C. The cheese-making procedures followed the steps described by Malheiros et al. [23], with some adaptations, using potassium chloride (0.02%; Rica Nata, Piracema, Brazil), cultures provided by natural low-fat yogurt (1.8%; Nestlé, São Paulo, Brazil), sodium chloride (0.8%; Sal Lebre, São Paulo, Brazil) and liquid coagulant (CHY-MAX^®^) as ingredients. The curds were placed in perforated and sterilized cylindrical molds that were kept at room temperature and turned every 1 h until the final dripping.

After production, the cheeses were kept refrigerated at 4 ± 1 °C for approximately 24 h until the physicochemical and sensory analyses were conducted. Samples were collected for physicochemical characterization, including moisture, by the gravimetric method. The cheese samples were pre-dried in a LV2000^®^ lyophilizer (Equipamentos Terroni Científicos, São Carlos, SP, Brazil). Ash (930.05), CP (981.10) and EE (920.39) were determined by the AOAC [13] methods, and yield was calculated as proposed by El-Gawad and Ahmed [24].

### 2.8. Sensory Analysis

Sensory evaluation was carried out through the application of a questionnaire in which five sensory attributes were assessed, namely, color, aroma, taste, texture and overall acceptability. Each attribute was assigned a score on a nine-point scale, as follows: dislike extremely (1), dislike very much (2), dislike moderately (3), dislike slightly (4), neither like nor dislike (5), like slightly (6), like moderately (7), like very much (8), and like extremely (9) (Appendix A).

The test was conducted with 103 untrained tasters who had been previously selected to include only dairy product consumers without allergies and who were interested in participating in sensory analysis. Of the 103 participants, 65% were female and 35% male; 86.3% were aged between 18 and 30 years, 9.7% between 31 and 40 years, 2.0% between 41 and 50 years and 2.0% between 51 and 60 years. Regarding the frequency of goat cheese consumption, 81% declared that they rarely consumed it, 15% consumed it occasionally and only 4% consumed it often.

The samples corresponding to the four treatments (0, 80, 160 and 240 g kg^−1^ of PKC inclusion) were offered in 50-mL cube-shaped cups with an average size of 3.4 cm^3^ to each of the tasters, together with crackers and mineral water. To avoid the mixing of tastes between samples, which might interfere with the sensory analysis, the tasters were advised to ingest a cracker and some water between tastings [25]. The samples were identified by codes with three random digits and were provided to the tasters in closed jars to maintain the sensory characteristics. The evaluation took place in the morning (between 9 a.m. and 12 p.m.).

### 2.9. Calculations 

The atherogenicity index (AI) = [12:0 + (4 × 14:0) + 16:0]/(Σn-6 + Σn-3 + ΣMUFA n-9), where 12:0 = lauric acid; 14:0 = myristic acid; 16:0 = palmitic acid; Σn-6 = sum of omega-6 polyunsaturated fatty acids; Σn-3 = sum of omega-3 polyunsaturated fatty acids; and ΣMUFA n-9 = sum of omega-9 monounsaturated fatty acids.

The thrombogenicity index (TI) = (14:0 + 16:0 + 18:0)/(0.5 × ΣMUFA) + (0.5 × Σn-6) + (3 × Σn-3) + (Σn-3/Σn-6), where 14:0 = myristic acid; 16:0 = palmitic acid; 18:0 = stearic acid; Σn-6 = sum of omega 6 fatty acids; Σn-3 = sum of omega-3 fatty acids; and ΣMUFA = sum of monounsaturated fatty acids.

The hypocholesterolemic-to-hypercholesterolemic fatty acid ratio (h:H) was evaluated and adapted in accordance with the method used by Bessa and Santos-Silva et al. [26,27]: h:H = (C18:1 cis9 + C18:2 n-6 + 20:4n-6 + C22:5n-3)/(C14:0 + C16:0).

Milk yield = [(0.93 F + C − 0.1) × 1.09 × 100]/(100 − M), where F is milk fat (%), C is casein (%) and M is moisture (%).

Non-fibrous carbohydrates (NFC) were calculated by the following equation [28]: NFC = 100 − (%NDFap + %CP + %EE + %ash).

The apparent digestibility of the nutritional components was estimated using the following formulae proposed by da Cruz et al. [29] for small ruminants:

(1) adCP = 0.7934 × CP% − 0.44
(2) adEE = 0.9107 × EE% − 0.33
(3) adNDFap = {0.7877 × (NDF − LIGNIN) + [1 − LIGNIN ÷ NDF)^0.85^]}
(4) adNFC = 0.9041 × NFC% − 3.22

where: adCP = apparent digestibility of crude protein; adEE = apparent digestibility of ether extract; adNDFap = apparent digestibility of neutral detergent fiber; and adNFC = apparent digestibility of non-fibrous carbohydrates.

After calculating the apparent digestibility of the nutritional components, the following formula was used to determine the total digestible nutrients (TDN): TDN = adCP + (adEE × 2.25) + adNDFap + adNFC.

### 2.10. Statistical Analysis 

Analyses of normality, variance, and regression were performed for the variables of cheese yield, proximate composition, fatty acid profile, AI, throTI and h:H ratio, with decomposition into linear and quadratic effects, considering a 5% probability level. For the analyses, the SAS statistical software version 9.2 (Statistical Analysis System, 2009) [30] was used.

The mathematical model below was applied:

Ŷij = μ + ILi + Ɛij,

where Ŷij = value observed in the plot that received treatment i in replicate j; μ = overall mean; ILi = fixed effect of PKC inclusion level i (i = 0, 80, 160 and 240 g kg^−1^); and Ɛij = random experimental error associated with each observation, with NID~(0, σ2) assumption.

The scores obtained in sensory analysis constituted a set of multivariate data that were arranged in a matrix (412 × 6) and interpreted using principal component analysis (PCA). For this analysis, SAS software version 9.2 (Statistical Analysis System) [30] was used with data centered on the mean.

## 3. Results

### 3.1. Intake and Milk Composition

The intakes of DM and CP decreased (*p* < 0.001) (Table 2).

The cheese fat and total solid contents exhibited a quadratic response, whereas the protein, lactose and casein contents decreased by 0.071 g day^−1^, 0.079 g day^−1^ and 0.002%, respectively (Table 2).

### 3.2. Milk Fatty Acid Profile

The addition of PKC to the diet induced a linear decrease in the caproic (6:0; *p* = 0.014), caprylic (8:0; *p* = 0.011) and capric (10:0; *p* = 0.003) short-chain fatty acids (SCFA). Each additional one-gram of PKC added to the diet resulted in reductions of 0.003, 0.012 and 0.003 mg 100 mg^−1^ in the concentrations of these SCFA, respectively (Table 3). Palmitic acid (16:0; *p* = 0.049) decreased linearly by 0.004 mg 100 mg^−1^ with each gram of PKC added to the diet, whereas the lauric (12:0; *p* = 0.012), myristic (14:0; *p* = 0.020), myristoleic (14:1; *p* = 0.014) and palmitoleic (16:1; *p* = 0.033) acids increased linearly (Table 3).

The diets had no effect on long-chain fatty acids (*p* > 0.05). The exception was linoleic acid (C18:2n6c), which showed a quadratic response (*p* = 0.002) to the addition of PKC, with a minimum value of 2.425 mg 100 mg^−1^ at the estimated PKC inclusion level of 156.1 g kg^−1^ of DM (Table 4).

Monounsaturated fatty acids increased linearly (*p* = 0.008) with the addition of PKC (Table 5). The ratio of PUFA:SFA showed a quadratic response (*p* = 0.022), with a minimum value of 0.052 mg 100 mg^−1^ occurring at the estimated PKC inclusion level of 118.72 g kg^−1^ DM. The thrombogenicity index decreased (*p* < 0.001) by 0.0018 with each gram of PKC added to the diet (Table 5).

### 3.3. Minas Frescal Cheese Quality

Cheese moisture and yield declined linearly (*p* < 0.05), by 0.025% and 0.01%, with each gram of PKC added to the diet, respectively (Table 6). The CP and EE contents of the cheese exhibited a quadratic behavior (*p* < 0.05), with maximum values of 43.48% and 46.87% DM achieved at the estimated PKC inclusion levels of 144.28 and 155.26 g kg^−1^ DM, respectively (Table 6).

### 3.4. Sensory Analysis

**Figure 1:**
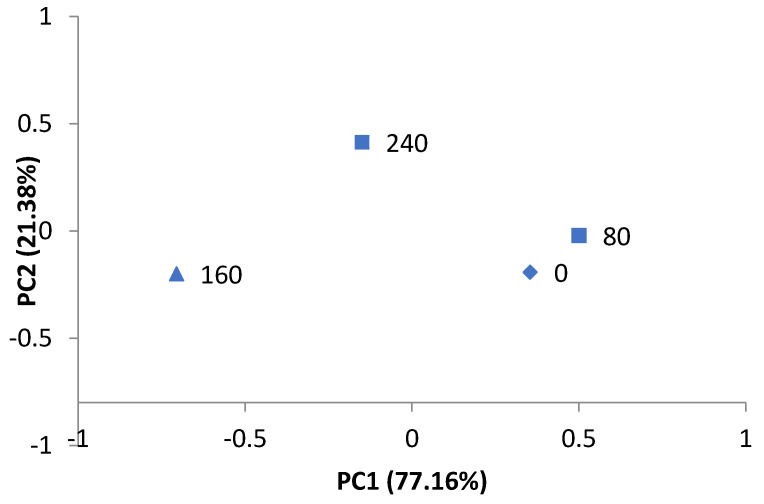
Principal components for the description of the sensory profile of cheese made of milk from goats fed palm kernel cake.

Principal component analysis revealed that the two principal components efficiently explained (98.54%) the variation and influence of the sensory parameters that were evaluated (Figure 1). Principal component 1 (PC1) was represented by the “taste” parameter, whereas principal component 2 (PC2) was represented by the “aroma” parameter (Table 7).

The graph representing the distribution of treatments from the two principal components shows a trend of separation according to the levels of inclusion of PKC in the lactating goats’ diet. The inclusion of higher levels of PKC was found to negatively affect the taste of the cheese. On the other hand, by observing PC2, we can establish that the cheese aroma became more pronounced with the inclusion of the ingredient.

The mean scores assigned to taste and aroma were 6.70 and 7.04, respectively.

## 4. Discussion

### 4.1. Milk Intake and Composition

The observed reduction of DM intake was due to the low acceptance of PKC by the goats. In the present experiment, the predominance of PKC in the orts explains the decreased intake. Silva et al. [31] observed similar behavior with increasing amounts of concentrate, with PKC in the orts of cull goats. 

Furthermore, Olawoye et al. [32] observed a reduction in the concentrate intake when PKC was added. In addition, the authors observed that to compensate for the total dry matter intake, goat kids ate a higher amount of silage. Rodrigues et al. [33] showed that the dry matter intake was reduced after the inclusion of 9.3% PKC in the dry matter of the total diet for goat kids. Those data can corroborate the PKC low-acceptance hypothesis.

Dry matter intake influences the intake of other dietary nutrients, as well as milk yield and milk components. Accordingly, NDF intake likely decreased as PKC was included, following DM intake. The fact that NDF is a substrate for rumen fibrolytic bacteria in the production of acetic acid, a fatty acid that serves as a substrate for the production of fat [34,35], explains the decrease in milk fat production with the increasing levels of PKC in the present experiment.

The decreasing DM intake also resulted in a lower intake of NFC. In the rumen environment, this component is degraded by bacteria and propionic acid is primarily produced. Propionic acid is the main precursor for the synthesis of glucose, which, in turn, is used in the production of lactose in the mammary gland [36].

Milk protein originates from the protein consumed in the diet, microbial protein synthesis and protein synthesis in the mammary gland [37]. In the present experiment, there was a decrease in the intakes of both DM and CP. The reduction of the substrate for microbial growth had a negative impact on the performance of the ruminal microbiota, which resulted in a smaller amount of protein, thus limiting the amount of absorbed protein and the substrate for its synthesis in the mammary gland [37].

The concentration of total solids considers all nutrients that are present in milk but not moisture. Therefore, the quadratic response shown by total solids was influenced by the fat concentration.

The current data could probably be extrapolated to other geographic realities or other goat breeds fed PKC as an ingredient of the diet. Olawoye et al. [38] evaluated the milk composition of West African dwarf goats fed PKC as a supplement in different ratios with the concentrate and reported increasing fat and protein contents. Considering the lower intake with PKC diets, the authors determined that the milk composition is probably due to body catabolism for milk production.

### 4.2. Fatty Acid Profile

Medium-chain fatty acids in milk are mainly derived from de novo synthesis. Thus, the observed reduction in MCFA (C6, C8, C10 and C16) is possibly due to the decreased intake of DM and other nutrients, which translates into a lower intake of substrates (e.g., acetate and β-hydroxybutyrate) needed for the de novo synthesis of MCFA [39]. Our findings corroborate those reported by Oliveira et al. [40], who also did not observe an increase in palmitic acid in milk from cows supplemented with increasing levels of PKC.

The increase in lauric (12:0) and myristic (14:0) acids is associated with their concentration in PKC, which is rich in these SFA (approximately 37.75% lauric acid and 19.51% myristic acid) [9,41].

The increased concentration of MUFA may have been due to a possible increase in Δ9-desaturase enzyme activity in the mammary gland, which led to the greater synthesis of C14:1 and C16:1 fatty acids [42,43]. This result is desirable, as the ingestion of MUFA brings benefits by reducing the total cholesterol content of blood, in addition to reducing insulin dependence in patients with type 2 diabetes [44].

Linoleic acid (C18:2 n6c) exhibited a quadratic response, which may have been caused by the presence of this fatty acid in the diets [45] or the desaturation process of trans-vaccenic fatty acid (C18:1 t11) that occurred in the mammary gland from the action of the delta-9 desaturase enzyme [43].

The quadratic behavior of the PUFA:SFA ratio was likely influenced by the quadratic response of linoleic acid (C18:2 n6c). This ratio influences the quality of the product with regard to human health, as it is correlated with the incidence of cardiovascular and chronic diseases. Values below 0.45 for this ratio are considered undesirable for human health [46]. However, the average PUFA:SFA value found in the present experiment (0.061) is within the range observed by Chen and Liu [47] in dairy products (0.02–0.175).

The atherogenicity and TI indices indicate the potential for platelet aggregation, which determines a greater or lesser likelihood of the onset of coronary heart disease. From the point of view of human health, it is recommended that AI and TI be less than 1.0 and 0.5, respectively [46]. The mean AI value in the present experiment was 2.64, which is close to the 2.34 and 2.60 values found by Idamokoro et al. [48], who evaluated the quality of milk from different goat breeds.

The thrombogenicity index is a function of MUFA and omega fatty acids. Thus, the linear decrease shown by TI was probably influenced by the total MUFA concentration, which grew linearly. The mean TI value in the present experiment was 2.94, which is within the range described by Chen and Liu [47] in goat milk (2.70–3.20).

### 4.3. Quality of Minas Frescal Cheese

Cheese quality and yield are influenced by numerous factors, the main ones being the composition and quality of milk and the manufacturing process [49].

Milk proteins, especially caseins, determine water retention in the cheese-making process [50]. In the present experiment, the CP and casein concentrations in the milk decreased linearly, influencing water retention. Consequently, the inclusion of PKC in the goats’ diet resulted in a cheese with lower moisture content.

Of the many factors that influence cheese yield, moisture is probably the most important [51]. Emmons [52] reported that a 1% increase in moisture in cheddar cheese increased its yield by 1.8%. Thus, the retention of solids and greater amounts of water from the milk in the manufacture of the cheese resulted in higher cheese yields.

The inclusion of PKC affects the goats’ acceptance of the diet [31]. At inclusion levels of around 12% [53], there is a decline in EE intake, which probably affects the composition of milk. The present findings corroborate this, as the milk fat concentration showed a quadratic response. Consequently, the behavior exhibited by the cheese fat was an expected result.

The fat concentration of milk inversely affects the protein concentration of cheese [54]. Accordingly, while the fat concentrations in milk and cheese showed concave curves, the cheese protein concentration curve was convex.

### 4.4. Sensory Analysis

Principal component analysis allows us to determine which variables, within a group of variables, are those that most influence a given parameter. In this way, the number of variables is reduced and the specific factors involved in the behavior of the parameter can be identified. The use of PCA showed that the sensory attributes with the greatest influence on the sensory evaluation of the cheese were taste and aroma.

Taste and aroma are two characteristics that directly affect the sensory quality of cheese [55]. In the present experiment, higher levels of PKC inclusion in the diet of the lactating goats negatively affected the taste of the cheese. On the other hand, the greater inclusion of PKC resulted in a more pleasant aroma for the tasters.

In this experiment, the inclusion of PKC in the lactating goats’ diet resulted in higher concentrations of MUFA. Increased MUFA concentrations in milk reduce cheese yield and increase the rate of lipolysis, consequently reducing the sensory acceptance parameters of the cheese [56]. This explains the observed results for the yield and taste of the Minas Frescal cheese produced in our experiment.

High concentrations of caproic, caprylic and capric fatty acids are associated with the greater intensity of “goat smell” in cheese [57,58]. The reduction of these fatty acids as a result of the inclusion of PKC explains the better rating for aroma by the evaluators in the present study. As observed by Colonna et al. [59], the fatty acid concentration is important in the sensory analysis of cheeses, considering that the “goaty” odor is viewed as undesirable by the panelists.

## 5. Conclusions

According to the results, the estimated inclusion of 80 g kg^−1^ palm kernel cake in the total diet of lactating goats is recommended, as it maintains the quality of the cheese made from the milk of these animals. Furthermore, the inclusion of palm kernel cake also improved the quality of the milk by reducing its thrombogenicity index.

Above the estimated level of 80 g kg^−1^ palm kernel cake in the total diet of lactating goats, the decreasing value of the parameters affects the productivity of the goats.

## Figures and Tables

**Table 1:** The proportion of ingredients and analyzed chemical composition of the diets including palm kernel cake.

Variable	Palm Kernel Cake (g kg^−1^)				Palm Kernel Cake
	0	80	160	240	
**Ingredient (g kg^−1^)**					
Maize silage	400.0	400.0	400.0	400.0	-
Palm kernel cake	0.0	80.0	160.0	240.0	-
Ground maize	320.0	260.0	200.0	140.0	-
Cottonseed meal	180.0	160.0	140.0	120.0	-
Maize germ	40.0	40.0	40.0	40.0	-
Soybean meal ^1^	50.0	50.0	50.0	50.0	-
Mineral supplement ^2^	10.0	10.0	10.0	10.0	-
**Chemical composition (g kg^−1^ DM)**					
Dry matter (g kg^−1^ as-fed)	681.5	683.9	686	688.8	923.4
Mineral matter	31.3	33.4	35.6	37.7	47.4
Crude protein	162.4	162.9	163.3	163.8	159.3
NDFap ^3^	337.8	369	398.8	428.6	617.9
ADFap ^4^	23.4	25.09	26.88	28.68	379.5
iNDF ^5^	140.8	151.2	161.6	17.2	202.7
pdNDF ^6^	196.9	217.7	237.2	256.6	415.2
Lignin	78.6	83.2	87.7	92.3	135.8
Ether extract	50.7	59.8	68.8	77.8	161.9
Non-fibrous carbohydrates	417.8	375	333.6	292.1	13.6
Total digestible nutrients	777.8	779.0	780.3	781.7	810.7

^1^ The soybean meal protein content was 48.33% DM basis; ^2^ provides per kilogram of active element: Calcium—183.00 g, Phosphorus—60.00 g, Potassium—28.00 g, Sulfur—16.00 g, Magnesium—20.00 g, Copper—250.00 mg, Cobalt—30.00 mg, Chromium—10.00 mg, Iron—250.00 mg, Iodine—70.00 mg, Manganese—1500.00 mg, Selenium—30.00 mg, Zinc—350,000.00 mg, Fluorine (max.)—600.00 mg. ^3^ Neutral detergent fiber corrected for ash and protein; ^4^ acid detergent fiber corrected for ash and protein; ^5^ indigestible neutral detergent fiber; ^6^ potentially digestible neutral detergent fiber.

**Table 2:** Intake of nutritional components and composition of milk from lactating goats fed diets with different levels of palm kernel cake.

	Palm Kernel Cake (g kg^−1^)					*p*-Value	
Variable	0	80	160	240	SEM ^1^	L ^2^	Q ^3^
**Intake (kg day^−1^)**							
Dry matter intake ^4^	1.68	1.68	1.25	0.84	0.78	<0.000	0.618
Crude protein intake ^5^	0.30	0.31	0.24	0.13	0.01	<0.000	0.614
**Milk composition (g day^−1^)**							
Fat ^6^	35.54	40.07	31.26	19.76	2.53	<0.000	0.005
Protein ^7^	32.05	30.29	24.48	16.7	2.21	<0.000	0.170
Lactose ^8^	40.13	38.15	32.92	22.67	3.11	<0.000	0.139
Total solids ^9^	116.18	117.19	96.01	64.08	8.25	<0.000	0.025
Casein (%) ^10^	2.87	2.93	2.61	2.39	0.09	0.0008	0.211

^1^ SEM—standard error of the mean; ^2^ L—linear; ^3^ Q—quadratic; significant at *p* < 0.05; ^4^ (DMI = 1.9 − 0.0042x. R^2^ = 0.99); ^5^ (CPI = 0.321 − 0.000742x. R^2^ =0.99) ^6^ (fat = 37.7081 + 0.005758x − 0.00056 × 2; R^2^ = 0.22); ^7^ (protein = 34.81 − 0.07104x. R^2^ =0.97); ^8^ (lactose = 43.44 − 0.07916x. R^2^ = 0.94); ^9^ (total solids = 121.73 + 0.01738x − 0.00108 × 2. R^2^ = 0.0948); ^10^ (casein = 2.966 − 0.00222x. R^2^ = 0.84).

**Table 3:** Short- and medium-chain fatty acids in milk from goats supplemented with different levels of palm kernel cake.

Variable	Palm Kernel Cake (g kg^−1^)				SEM ^1^	*p*-Value	
	0	80	160	240		L ^2^	Q ^3^
**Fatty acid profile (mg 100 mg^−1^)**							
Butyric acid (C4:0)	1.451	1.433	1.342	1.367	0.062	0.584	0.879
Caproic acid (C6:0) ^4^	2.074	2.000	1.65	1.512	0.093	0.014	0.842
Caprylic acid (C8:0) ^5^	2.409	2.223	1.722	1.435	0.152	0.011	0.946
Capric acid (C10:0) ^6^	7.785	6.646	4.903	3.849	0.535	0.003	0.959
Lauric acid (C12:0) ^7^	3.36	4.812	5.703	6.184	0.423	0.012	0.509
Myristic acid (C14:0) ^8^	8.301	8.936	9.779	10.044	0.229	0.020	0.721
Myristoleic acid (C14:1) ^9^	0.093	0.122	0.129	0.142	0.009	0.014	0.781
Pentadecanoic acid (C15:0)	0.593	0.694	0.679	0.704	0.045	0.485	0.708
Palmitic acid (C16:0) ^10^	25.364	25.163	23.851	23.06	1.58	0.049	0.723
Palmitoleic acid (C16:1) ^11^	0.527	0.935	0.815	0.917	0.107	0.033	0.985

^1^ SEM—standard error of the mean; ^2^ L—linear; ^3^ Q—quadratic; significant at *p* < 0.05); ^4^ (C6:0=2.114−0.00255x. R^2^ = 0.94); ^5^ (C8:0=2.4609−0.00428x R^2^ = 0.97); ^6^ (C10:0=7.828−0.01694x. R^2^ = 0.93); ^7^ (C12:0=3.61+0.012x. R^2^ = 0.95); ^8^ (C14:0=8.3544+0.0076x. R^2^ = 0.97); ^9^ (C14:1=0.08991+0.00022x. R^2^ = 0.95); ^10^ (C16:0=25.588−0.004325x. R^2^ = 0.93); ^11^ (C16:1=0.5839+0.002381x. R^2^ = 0.75).

**Table 4:** Long-chain fatty acids in milk from goats supplemented with different levels of palm kernel cake.

Variable	Palm Kernel Cake (g kg^−1^)				SEM ^1^	*p*-Value	
	0	80	160	240		L ^2^	Q ^3^
**Fatty acid profile (mg 100 mg^−1^)**							
Heptadecanoic acid (C17:0)	0.465	0.018	0.026	0.150	0.078	0.161	0.064
Stearic acid (C18:0)	11.877	12.779	13.092	12.314	0.455	0.718	0.409
Vaccenic acid (C18:1 t11)	3.215	3.245	2.070	2.739	0.496	0.598	0.771
Oleic acid (C18:1 n9c9)	19.531	20.604	23.686	24.36	1.019	0.067	0.92
Linoleic acid (C18:2n6c) ^4^	3.634	2.748	2.781	2.766	0.151	0.002	0.004
Arachidic acid (C20:0)	0.238	0.254	0.250	0.266	0.016	0.617	0.999
Linolenic acid (C18:3n3)	0.141	0.114	0.208	0.228	0.033	0.221	0.087
Rumenic acid (C18:2c9t11)	0.609	0.556	0.503	0.594	0.056	0.229	0.771
Arachidonic acid (C20:4n6)	0.212	0.189	0.190	0.201	0.013	0.918	0.096
Eicosapentaenoic acid (C20:5n3)	0.025	0.017	0.022	0.023	0.003	0.904	0.476

^1^ SEM—standard error of the mean; ^2^ L—linear; ^3^ Q—quadratic; significant at *p* < 0.05; ^4^ ($C18:2n6c=3.6451-0.01561x+0.00005x^{2}$. R^2^ = 0.46).

**Table 5:** Fatty acid profile of milk from goats supplemented with different levels of palm kernel cake.

Variable	Palm Kernel Cake (g kg^−1^)				SEM ^1^	*p*-Value	
	0	80	160	240		L ^2^	Q ^3^
**Fatty acid profile (mg 100 mg^−1^)**							
**PUFA**	4.384	3.418	3.491	3.589	0.16	0.090	
**MUFA ^4^**	24.139	25.684	27.659	29.124	0.725	0.008	0.974
**SFA**	64.347	65.358	63.315	61.620	1.787	0.092	0.284
**PUFA:SFA ^5^**	0.068	0.052	0.055	0.067	0.003	0.981	0.022
**Total**	92.871	94.459	94.465	94.22	1.54	0.182	0.167
**n-6**	3.634	2.748	2.781	2.766	0.151	0.057	0.119
**n-3**	0.141	0.114	0.208	0.228	0.033	0.221	0.087
**n6:n3**	35.176	25.424	25.599	14.040	3.657	0.064	0.899
**CLA**	0.609	0.556	0.503	0.594	0.056	0.862	0.568
**AI**	2.658	2.872	2.614	2.431	0.138	0.490	0.513
**TI ^6^**	3.175	3.214	2.950	2.405	0.124	0.000	0.023
**h:H**	0.622	0.59	0.664	0.979	0.084	0.511	0.859

^1^ SEM—standard error of the mean; ^2^ L—linear; ^3^ Q—quadratic; significant at *p* < 0.05); PUFA—polyunsaturated fatty acids; ^4^ MUFA—monounsaturated fatty acids =24.1122+0.02116x, R^2^ = 0.99; SFA—saturated fatty acid; ^5^ PUFA:SFA—ratio of polyunsaturated to saturated fatty acids $=0.06775-0.00026x+0.000001095x^{2}$, R^2^ = 0.98; Total—total fatty acids; n-6—omega-6 fatty acids; n-3—omega-3 fatty acids; CLA—sum of conjugated linoleic acids; AI—atherogenicity index; ^6^ TI—thrombogenicity index =3.2476−0.00182x, R^2^ = 0.84); h:H—ratio of hypocholesterolemic to hypercholesterolemic fatty acids.

**Table 6:** Chemical composition and yield of Minas Frescal cheese produced from milk from goats supplemented with different levels of palm kernel cake.

	Palm Kernel Cake (g kg^−1^)				SEM ^1^	*p*-Value	
Variable	0	80	160	240		L ^2^	Q ^3^
**% of dry matter**							
Moisture ^4^	63.50	54.49	57.76	54.90	1.02	0.004	0.123
Crude protein ^5^	44.62	44.65	42.58	44.40	0.28	0.146	0.041
Ether extract ^6^	42.21	46.08	46.76	45.89	0.53	<0.000	0.002
Mineral matter	7.02	6.11	6.63	6.25	0.14	0.110	0.279
**kg of cheese per 100 kg of milk**							
Yield ^7^	20.00	16.18	17.68	16.41	0.43	0.003	0.129

^1^ SEM—standard error of the mean; ^2^ L—linear; ^3^ Q—quadratic; (significant at *p* < 0.05); ^4^ ($\mathrm{moisture}=60.5211-0.02537x.{\;R}^{2}=0.44$); ^5^ ($\mathrm{crude}\;\mathrm{protein}=44.92-0.0202x+0.000070x^{2}$. R^2^ = 0.27); ^6^ (ether extract$=42.29+0.059x-0.00019{\;x}^{2}$. R^2^ = 0.45); ^7^ (yield=18.7311−0.01037x. R^2^ = 0.44).

**Table 7:** Estimates of principal components related to the sensory evaluation of Minas Frescal cheese, produced from the milk of goats supplemented with different levels of palm kernel cake.

PC_i_	Eigenvalue	Proportion of Variance (%)	Cumulative Proportion (%)	Weighting Coefficient				
				Color	Aroma	Taste	Texture	OA
**PC_1_**	0.299	77.160	77.160	−0.009	0.203	**0.700**	0.307	0.613
**PC_2_**	0.083	21.380	**98.540**	0.050	**0.903**	−0.360	0.227	−0.002
PC_3_	0.006	1.460	100.000	0.426	−0.330	−0.261	0.800	0.010
PC_4_	0.000	0.000	100.000	0.903	0.105	0.151	−0.388	0.000
PC_5_	0.000	0.000	100.000	0.000	0.150	0.539	0.249	−0.790

PC—principal component; OA—overall acceptance.

## Data Availability

The study did not report any data.

## Appendix A

SENSORY EVALUATION OF GOAT CHEESE				
Name: ______________________________________________________				
Gender:	F □	M □	Age:__________	Date:___/___/______

You are taking part in a scientific study on the “Sensory Analysis of Goat Cheese”. Please be as honest as possible in your answers, as they are extremely important to the success of this study. We thank you for your participation and collaboration.

How often do you consume goat cheese?

(  ) Rarely (2 to 5 times in a year)

(  ) Occasionally (More than 5 times in a year)

(  ) Often (More than once per month)

See how you should rate the cheese characteristics:

	**ATTRIBUTE**	
9	Like extremely	9
8	Like very much	8
7	Like moderately	7
6	Like slightly	6
5	Neither like nor dislike	5
4	Dislike slightly	4
3	Dislike moderately	3
2	Dislike very much	2
1	Dislike extremely	1

**Sample 077**		**Sample 473**		**Sample 164**		**Sample 521**	
Attribute	Score	Attribute	Score	Attribute	Score	Attribute	Score
Color		Color		Color		Color	
Aroma		Aroma		Aroma		Aroma	
Taste		Taste		Taste		Taste	
Texture		Texture		Texture		Texture	
Overall acceptance		Overall acceptance		Overall acceptance		Overall acceptance	

List the samples in the order of your preference for taste and aroma:

	**1st Place**	**2nd Place**	**3rd Place**	**4th Place**
Taste				
Aroma				
